# Virtual monoenergetic micro-CT imaging in mice with artificial intelligence

**DOI:** 10.1038/s41598-022-06172-0

**Published:** 2022-02-11

**Authors:** Brent van der Heyden, Stijn Roden, Rüveyda Dok, Sandra Nuyts, Edmond Sterpin

**Affiliations:** 1 Department of Oncology, Laboratory of Experimental Radiotherapy, KU Leuven, Leuven, Belgium; 2 Institut de Recherche Expérimentale Et Clinique, Molecular Imaging Radiotherapy and Oncology Lab, UCLouvain, Brussels, Belgium

**Keywords:** Cancer, Mathematics and computing, Physics

## Abstract

Micro cone-beam computed tomography (µCBCT) imaging is of utmost importance for carrying out extensive preclinical research in rodents. The imaging of animals is an essential step prior to preclinical precision irradiation, but also in the longitudinal assessment of treatment outcomes. However, imaging artifacts such as beam hardening will occur due to the low energetic nature of the X-ray imaging beam (i.e., 60 kVp). Beam hardening artifacts are especially difficult to resolve in a ‘pancake’ imaging geometry with stationary source and detector, where the animal is rotated around its sagittal axis, and the X-ray imaging beam crosses a wide range of thicknesses. In this study, a seven-layer U-Net based network architecture (vMonoCT) is adopted to predict virtual monoenergetic X-ray projections from polyenergetic X-ray projections. A Monte Carlo simulation model is developed to compose a training dataset of 1890 projection pairs. Here, a series of digital anthropomorphic mouse phantoms was derived from the reference DigiMouse phantom as simulation geometry. vMonoCT was trained on 1512 projection pairs (= 80%) and tested on 378 projection pairs (= 20%). The percentage error calculated for the test dataset was 1.7 ± 0.4%. Additionally, the vMonoCT model was evaluated on a retrospective projection dataset of five mice and one frozen cadaver. It was found that beam hardening artifacts were minimized after image reconstruction of the vMonoCT-corrected projections, and that anatomically incorrect gradient errors were corrected in the cranium up to 15%. Our results disclose the potential of Artificial Intelligence to enhance the µCBCT image quality in biomedical applications. vMonoCT is expected to contribute to the reproducibility of quantitative preclinical applications such as precision irradiations in X-ray cabinets, and to the evaluation of longitudinal imaging data in extensive preclinical studies.

## Introduction

Micro Computed Tomography (CT) imaging is currently indispensable in preclinical small animal research. The characterization of preclinical models by micro-CT imaging is key to improve the reproducibility of clinical applications in many biomedical research areas. Preclinical micro-CT imaging has been widely adopted in biomedical applications already, e.g. to evaluate the tumor volume by image segmentation^1^, to monitor disease states^2,3^, to assess the degree of muscle atrophy^4,5^, or to perform dose calculations in preclinical image-guided radiotherapy research^6,7^.

In past years, dedicated image-guided small animal irradiation cabinets have been developed commercially to facilitate the preclinical radiotherapy workflow in cancer research^7^. Currently, two companies are actively manufacturing these preclinical imaging and irradiation cabinets: Precision X-ray Incorporated (PXi, North Brandford, CT) with their X-RAD SmART system^8^, and Xstrahl Inc. (Xstrahl, Suwanee, GA) with their SARRP system^9^. These cabinets are both equipped with an X-ray tube and a flat-panel imaging detector for submillimeter micro cone-beam CT (CBCT) imaging. However, the imaging setup is manufactured differently between them. The X-RAD SmART system follows a conventional CBCT imaging setup, wherein the source and the detector rotate simultaneously around a stationary table in 360 degrees. Here, the imaging setup is rotated around the longitudinal axis, and thus the shortest beam path through the geometry being imaged. The Xstrahl SARRP system adopts a different so-called table-rotating ‘pancake’ geometry^10^ with stationary source and detector, where the prone-positioned animal is rotated around its sagittal axis, and the imaging beam traverses a wide range of thicknesses through the geometry being imaged^11^. Therefore, the mechanical adoption of a ‘pancake’ geometry could give rise to additional artifacts in the CBCT image reconstruction, such as beam hardening. This work mainly aims to resolve CBCT image artifacts that emerge from the so-called ‘pancake’ imaging geometry.

Micro CBCT image artifacts are mainly introduced by cardiorespiratory motion^12^, contaminating (in)elastic photon scatter distributions^13^, and beam hardening^14,15^. Photon scatter leads to a contaminating particle fluence measured by the detector panel, but in preclinical low-energy X-ray imaging, it is primarily beam hardening that introduces a degraded image quality. The energy-dependence of material-specific linear attenuation coefficients and the polyenergetic nature of the incident imaging beam cause the X-ray spectrum to harden in function of penetration depth. A hardened X-ray spectrum becomes more penetrating and is therefore introducing an error in the beam intensity measurements. A CBCT image reconstruction without beam hardening correction yields a degraded image quality across uniform volumes, expressed as non-uniform CT numbers, which negatively affects the dosimetric quantities^16^, but also the accuracy of CBCT image feature extraction for which the radiation cabinets could potentially be used^4,17^.

Several studies have already indicated that tissue identification is a source of errors in preclinical kV dose calculations due to the strong dependence of the photo-electron absorption cross sections on the effective atomic number of the tissues^16,18^. Therefore, beam hardening correction algorithms and alternative solutions have been suggested in literature to obtain a desirable CT image quality. First, Kachelrieß et al.^19^ presented an empirical water-based correction algorithm to correct CT cupping artifacts by nonlinearities in the projection data of conventional CT geometries. This empirical correction algorithm aims at linearizing the attenuation data using a polynomial correction in the image-domain. Here, the polynomial coefficients are determined from calibration scans of different-sized homogeneous cylindrical phantoms. However, this correction algorithm assumes a conventional CBCT imaging setup wherein the source and the detector rotate simultaneously around the longitudinal axis of the object being imaged. Therefore, this correction workflow is not directly applicable to the SARRP imaging setup without major algorithmic modifications. Next, two empirical CT correction algorithms were published to correct for higher order beam hardening artifacts in the imaging domain that originate from a mixture of materials, such as water, bone, and iodine^14,15^. Although some of the empirical corrections eliminate the need of a priori information, their practice in online preclinical irradiations is limited by (i) the time needed for a series of image reconstructions, and by (ii) the computationally intensive optimization procedure which is essential to produce beam hardening-corrected images. It is relevant to note that most empirical beam hardening correction algorithms require the prior implementation of a water precorrection^19^ algorithm to correct for cupping. However, water precorrection algorithms are mainly introduced for conventional CT setups, and not ‘pancake’ geometries. Alternatively, Yang et al.^10^ suggested a different solution to introduce an animal inclination angle of approximately 30 degrees in the pancake geometry. The inclination angle improved the transmission uniformity of the imaging beam, and therefore it approaches a conventional CBCT imaging setup. However, the installation of an inclination device requires additional efforts to secure stationary and reproducible animal positioning during fractionated irradiation experiments.

Recently, the emergence of Artificial Intelligence (AI) applications has caused a paradigm shift in clinical cone-beam CT imaging that suffers from contaminating X-ray scatter that give rise to a reduced the image quality^20,21^. Several effective AI applications resolved the problem of X-ray scatter accurately, and they even accelerated the calculation of adaptive radiotherapy plans on clinical CBCT images^22,23^. Clinical CBCT systems typically operate at higher X-ray energies (e.g. 120 kVp), which are more sensitive to (in)elastic X-ray scatter than lower-energy preclinical µCBCT systems (e.g. 60 kVp). The low-energy X-ray imaging beams adopted in preclinical µCBCT mainly interact inside the animals with photo-electric absorption, which gives rise to beam hardening. Particularly the ‘pancake’ geometry-based systems are subject to beam hardening, because the imaging beam crosses a wide range of animal thicknesses.

In analogy with the clinical AI corrections, this work presents an AI solution to transform polyenergetic X-ray projections to virtual monoenergetic X-ray projections, solely based on a Monte Carlo simulated training dataset. It is hypothesized that the µCBCT image reconstruction based on AI-corrected virtual monoenergetic X-ray projections removes artifacts caused by the polyenergetic nature of the X-ray imaging beam.

## Materials and methods

### Monte Carlo simulation model

A Monte Carlo simulation model of the SARRP µCBCT platform was developed in TOPAS^24^ (Version 3.6). The µCBCT simulation model was made of three major components: the virtual mono-or polyenergetic X-ray source, the digital phantom objects being imaged, and the flat-panel imaging detector. The “g4em-standard_opt4” physics list was adopted in the TOPAS model for the simulation of virtual mono- and polyenergetic X-ray projections. The lower-edge particle production cut was set to 5.0 keV, and to improve calculation times, no secondary electrons were transported inside the digital phantoms being imaged. The source-to-origin distance of the mono- and polyenergetic X-ray sources was 353.4 mm, and the detector-to-origin distance of the flat-panel imaging detector was 271.0 mm. The source and detector distances were determined according to the dedicated SARRP calibration procedures.

A virtual monoenergetic imaging energy of 35.0 keV, above the K-absorption edge of iodine (i.e., 33.2 keV), was adopted in the simulation procedure. The SARRP X-ray spectrum was calculated in an open-access Python package for the accurate modelling of X-ray spectrum named SpekPy^25^. The 60 kVp X-ray spectrum was simulated in SpekPy with 0.8 mm beryllium of inherent filtration, 1.0 mm aluminum of spectral filtration, and an X-ray tube anode angle of 20 degrees. A gaussian focal spot size of 0.6 mm was defined in the TOPAS model for the mono-and polyenergetic X-ray sources.

A realistic flat panel model with an active area of 176 × 128 mm^2^ was simulated as SARRP imaging detector. The detector was modelled with a pixel spacing of 0.5 × 0.5 mm^2^, and included a protective entrance carbon plate of 1 mm, an air gap of 3 mm, a cesium iodine scintillator of 600 μm, a glass substrate of 1 mm (for the photodiodes), and an aluminum back plate of 1.5 mm.

### The creation of reference mouse geometries

The 3D whole body mouse atlas DigiMouse was used as reference simulation and material geometry throughout this work^26^. Two pre-processing steps were performed to accelerate Monte Carlo simulation times, and to create a material phantom that is interpretable by our simulation model. First, the original DigiMouse µCT and its corresponding annotated atlas (0.1 × 0.1 × 0.1 mm^3^) were down sampled by a factor of two. Then, one template DigiMouse material phantom was created manually by assigning a list of 15 tissue compositions to the annotated atlas volume^27^. Further action in the data generation process includes the data collection of a retrospective µCBCT dataset^28,29^ of nine 6–7-week-old female nu/nu Naval Medical Research Institute (NMRI) mice (Janvier Labs, France). The retrospective mice imaging dataset was obtained from an initial experiment that was approved by the Ethical committee Animal Experimentation of KU Leuven (P163/2017; approval date 29 September 2017). The µCBCT dataset was collected to define mouse-specific reference geometries for simulation by deformable image registration in Elastix^30^. First, the down sampled DigiMouse µCT was weakly deformed by affine and Bspline transformations to each of the nine retrospective µCBCT datasets. A weak deformation (i.e., Elastix “FinalGridSpacingInVoxels” parameter equal to [16.0 16.0 16.0]) was adopted to avoid abnormal mouse anatomies due to overfitting errors in the deformation process of two physically unrelated geometries. Next, the transformation matrices were copied and applied to the template DigiMouse material phantom to obtain nine mouse-specific digital reference geometries that will be used for simulation.

### The simulation of projection data

The nine mouse-specific digital reference geometries are adopted for the simulation of projection pairs (60 kVp–35 keV). For each of the nine digital mouse phantoms, 105 projection pairs were simulated in our Monte Carlo model. Here, a randomly sampled degree of variation is included in the simulation of the X-ray projections. The variation is included in the definition of the random anisotropic voxel size [0.17–0.29 mm], the three-dimensional digital phantom position [± 10 mm], the X-ray projection angle [0–359 degrees], the elemental variation^16^ in the tissue composition of ± 5%, and finally a mass density variation of ± 5%. Furthermore, the dataset was augmented through the simulation of mathematical cylinders varying in diameter, position, orientation, and tissue composition. Here, 945 projection pairs were simulated, which leads to a total number of 1890 projection pairs for model learning. Finally, an image rescaling from 352 × 56 pixels to 704 × 51 pixels was applied on all simulated projection pairs prior to network training. The network performance is assessed with the percentage error metric (100% $\cdot$ |predicted—reference|/reference).

### vMonoCT-network architecture

**Figure 1:**
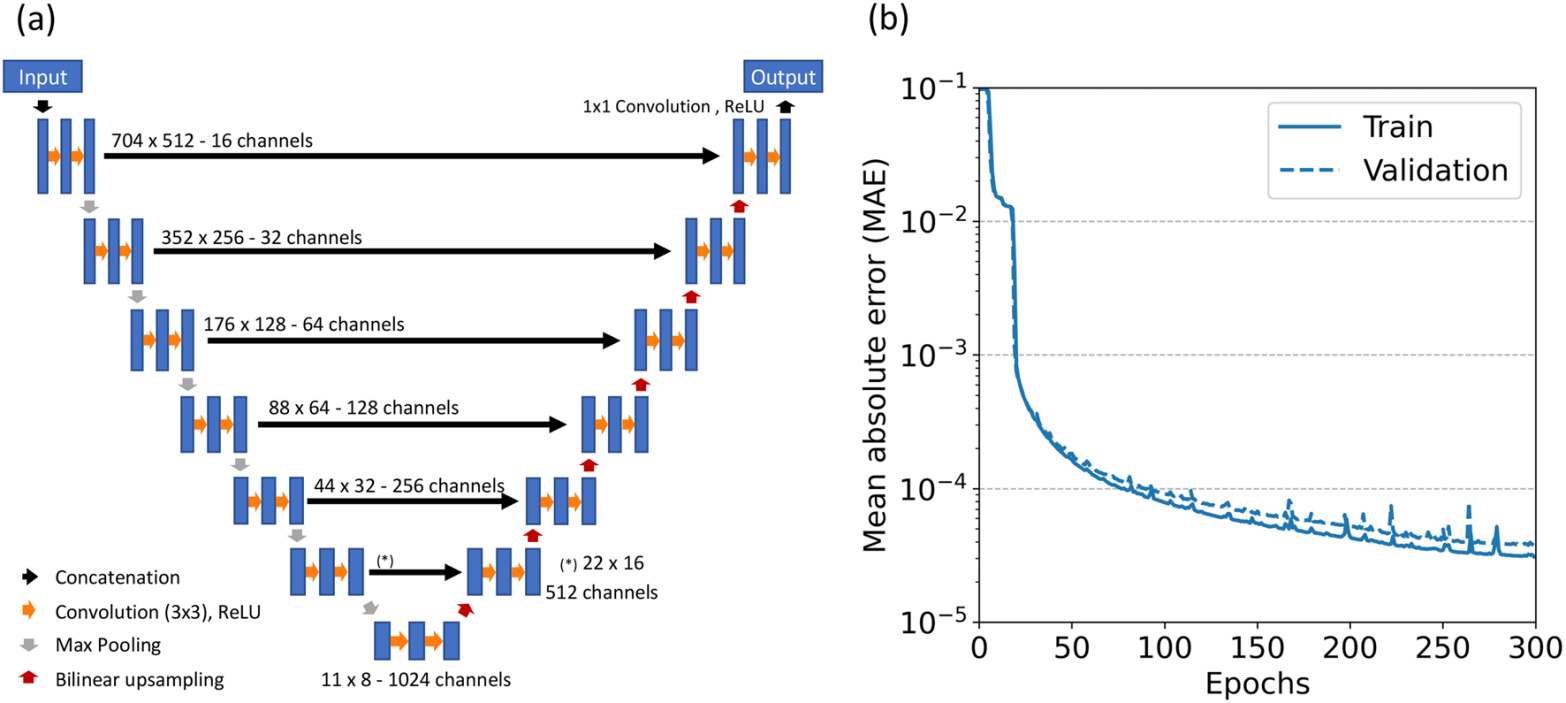
**a** The virtual monoenergetic micro cone-beam CT (vMonoCT) network architecture. **b** The mean absolute error (MAE) loss function of the vMonoCT network plotted against the number of epochs for the training and validation datasets.

A range of monoenergetic and polyenergetic X-ray projection pairs were calculated with the Monte Carlo simulation model. Here, a U-Net based architecture is adopted to predict virtual monoenergetic X-ray projections from polyenergetic X-ray projections. The vMonoCT network architecture (Fig. 1a) consists of 7 layers, existing of a contraction and an expansion path, where the contraction layer output is concatenated in the channel dimension with the expansion layer input.

Every block of the contraction path applies successive 3 × 3 convolution layers followed by a rectified linear unit (ReLu). The spatial resolution in every dimension is halved every block by a 2 × 2 max pooling layer. The number of feature maps after each block doubles along the contraction path from 16 to 1024. The difference between the contraction and the expansion path is that the max pooling operations in the contraction path are replaced by bilinear up sampling operations in the expansion path. The Adam optimizer was employed as stochastic training optimizer with an initial learning rate of 1E-5 and parametrized with ${\beta }_{1}$ = 0.900, ${\beta }_{2}$ = 0.999. The fixed batch size was set to 18, the number of epochs to 300, and the mean absolute error (MAE) was adopted as loss function to train the convolutional neural network. No batch-normalization was applied to prevent unwanted scaling of the predicted virtual monoenergetic X-ray projections. vMonoCT was trained on the Google cloud Platform, in a virtual environment with an Intel® Xeon® CPU @ 2.20 GHz and a 16 GB T4 NVIDIA® GPU. vMonoCT was implemented in Keras (v. 2.4.3) with Tensorflow as backend (v. 2.2.0). Here, 1512 projection pairs were used for training (= 80%), and 378 projection pairs were used for testing (= 20%).

### µCBCT imaging protocol and reconstruction

The vMonoCT network performance was assessed in real mouse experiments on projection data directly obtained from the SARRP µCBCT imaging platform. The vMonoCT ability to remove image artifacts was first evaluated on a frozen cadaver in absence of breathing artifacts, and then on five retrospective mouse projection datasets. In the µCBCT acquisition of the frozen cadaver, an Eppendorf® tube filled with water was attached below the animal bed to evaluate image uniformity in a homogeneous volume-of-interest according to the uniform normalized absolute average deviation (UNAAD) metric^31^, where ${Y}_{i}$ is the individual voxel intensity value, and N is the total number of voxels within the volume-of-interest used for the calculation of the mean image intensity $\overline{Y }$. Larger UNAAD values imply a more uniform µCBCT image reconstruction.

1 $$\mathrm{UNAAD}= 100 -\frac{100}{\mathrm{N}\cdot \overline{\mathrm{Y}} } \sum_{\mathrm{i}=1}^{\mathrm{N}}\left|{\mathrm{Y}}_{\mathrm{i}}-\overline{\mathrm{Y} }\right|$$

At the time of writing, the closed-source SARRP pilot reconstruction software provides arbitrary reconstruction values that are scaled internally to match the 16-bit signed integer output format, which limits the direct comparison of multiple image reconstructions. Therefore, an in-house developed FDK reconstruction toolkit was developed using the open-source C++ RTK software^32^. The corrected and uncorrected image projections were reconstructed in a 256 × 256 × 600 image matrix with 0.2 × 0.2 × 0.2 mm^3^ voxel spacing. The 60 s µCBCT scan protocols were performed with 1 mm of aluminum spectral filtration, a tube voltage of 60 kVp, and a beam current of 0.8 mA^33^.

## Results

### vMonoCT model performance

A computation time of 6 h and 34 min was required to train the vMonoCT network architecture for 300 epochs. Figure 1b depicts the logarithmic MAE loss curve associated with the training of the vMonoCT network architecture. The prediction time of the virtual monoenergetic projections on the test dataset was 21.3 s, and the MAE was 2.6E-3 ± 1E-3 (± 1 standard deviation). Furthermore, the prediction time of the frozen cadaver dataset was 19.0 s, and the prediction time of the five retrospective mouse datasets was equal to 18.8 ± 0.2 s (52.2 ms per projection).

**Figure 2:**
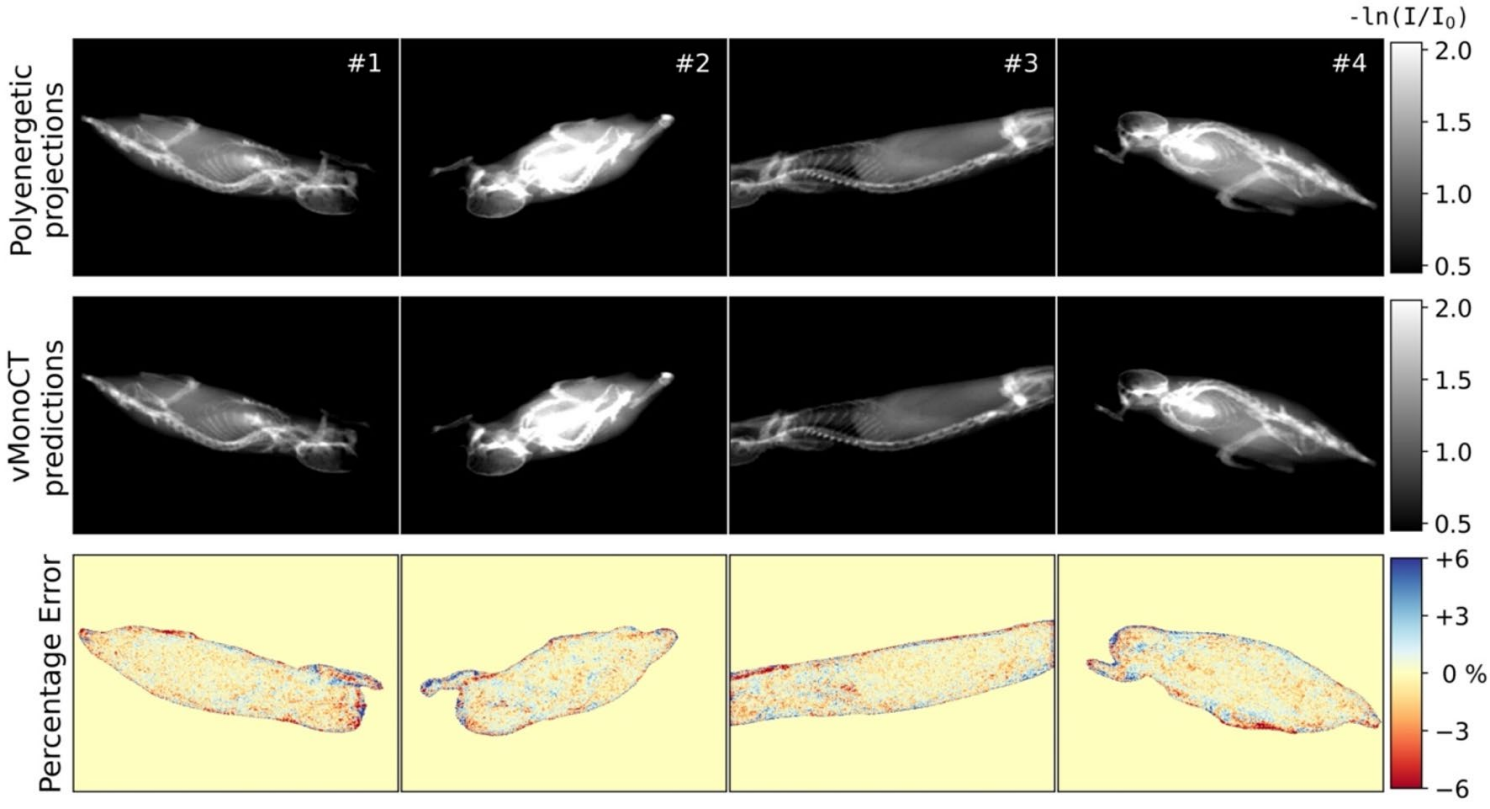
The percentage error maps of four randomly selected projections calculated between the simulated polyenergetic projections as network-input, minus the vMonoCT predicted projections [-ln(I/I_0_)].

Figure 2 compares four randomly picked polyenergetic projections of the test dataset with the vMonoCT predictions. The percentage error of the four reported projections in respective order was equal to 1.6 ± 1.9%, 1.6 ± 2.0%, 1.5 ± 1.8%, and 1.6 ± 1.9%. Overall, the percentage error calculated for the entire test dataset was 1.7 ± 0.4%. All errors excluded the pixels in the X-ray projection that represented air trajectories. The largest errors were observed near sharp material transitions, e.g., air and the animal body. A Wilcoxon signed-ranks test (*p* = 0.44) indicated that no large prediction loss was observed for longer imaging beam paths, compared to smaller paths, through the animal, e.g. animal #2 in Fig. 2.

### vMonoCT applied to real µCBCT projections

**Figure 3:**
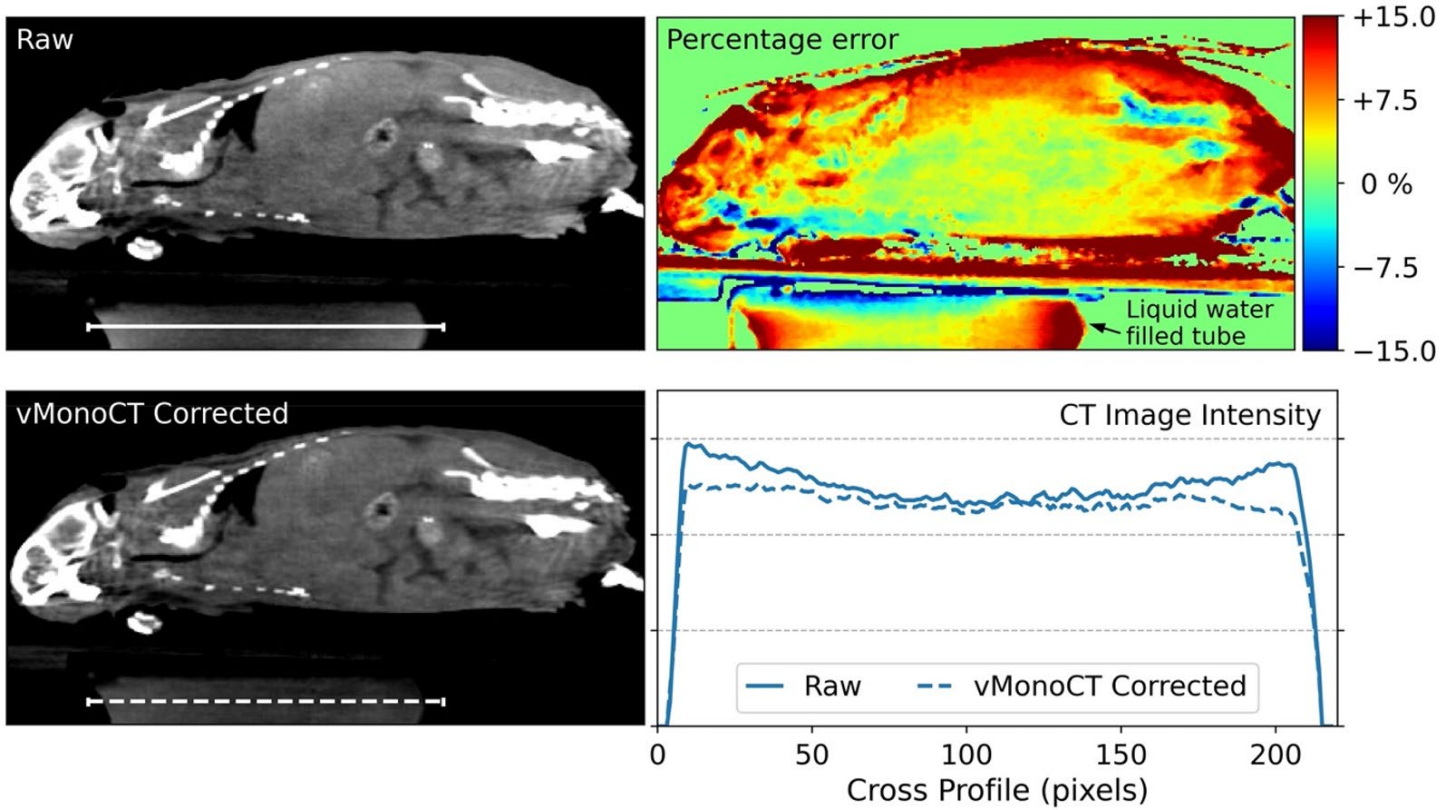
The raw and vMonoCT corrected SARRP µCBCT image reconstruction of a frozen mouse cadaver, including the imaging of a liquid water filled tube to evaluate cupping. The percentage error map is calculated between the raw image, and minus the vMonoCT corrected µCBCT image. An image cross profile was plotted through the liquid water filled tube for both reconstructions.

The center slice of the raw and vMonoCT corrected µCBCT reconstructions of the frozen cadaver are shown in Fig. 3. For both µCBCT image reconstructions, a cross profile was plotted through the liquid water filled tube that was attached below the animal bed. The UNAAD metric was calculated for both image reconstructions. The vMonoCT corrected image reconstruction showed a higher UNAAD value (= 97.0) than the raw image reconstruction (= 94.3) in the water tube, meaning that a higher image uniformity was obtained by the AI method. Here, it was also illustrated that the vMonoCT corrected image reconstruction shows less cupping than the raw image reconstruction. An additional two-dimensional percentage error plot was added to evaluate peripheral beam hardening artifacts.

**Figure 4:**
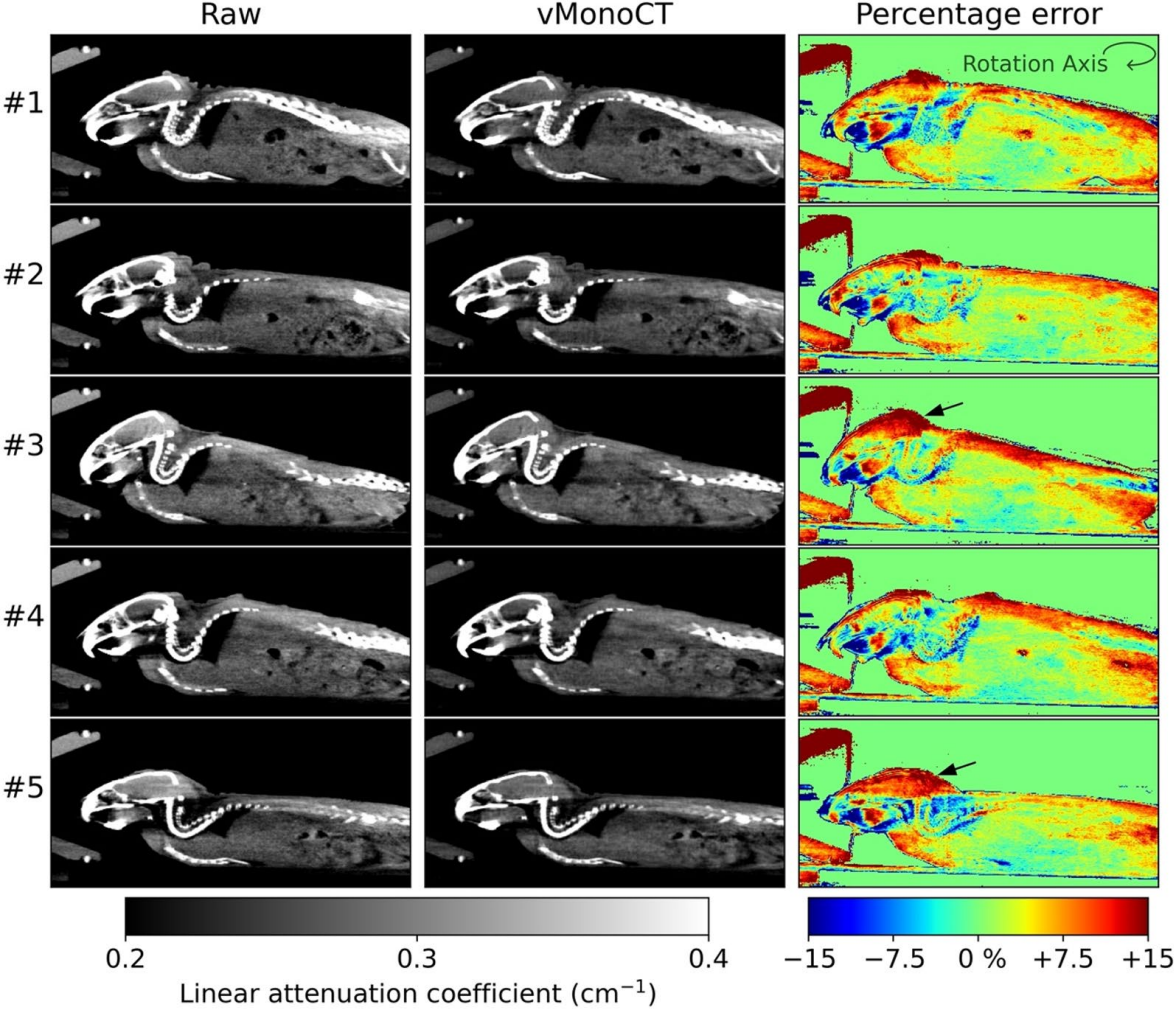
The raw and vMonoCT corrected SARRP µCBCT image reconstructions of a retrospective dataset existing of five mice. The percentage error map is calculated between the raw images, and minus the vMonoCT corrected µCBCT images.

Next, the vMonoCT network architecture was applied and tested on a retrospective dataset of five mice. Figure 4 illustrates the central slices of the five µCBCT image reconstructions, and the percentage error maps. The table rotation axis of the ‘pancake’ imaging geometry is indicated by the circular arrow in the top right corner of Fig. 4, and the two black arrows (Mouse #3 and #5) indicate seriously pronounced gradient errors in the cranial region up to 15%.

## Discussion

Beam hardening is a challenging problem which arises in preclinical µCBCT imaging. It introduces image artifacts caused by the polyenergetic nature of the X-ray spectra. Especially in ‘pancake’ imaging geometries, beam hardening is difficult to be resolved by time-efficient software correction methods. In this work, a convolutional neural network (vMonoCT) was presented to predict virtual monoenergetic X-ray projections from a polyenergetic input. vMonoCT was trained on a diversly augmented Monte Carlo simulation dataset of a digital anthropomorphic reference phantom (DigiMouse).

The network showed a good prediction accuracy of virtual monoenergetic projections on the test dataset, and vMonoCT was evaluated on real µCBCT projection data from the SARRP research cabinet (Figs. 3 and 4). Here, difference maps were depicted to illustrate the difference between the raw image reconstruction and the vMonoCT-based image reconstruction. It should be noted that the percentage differences between the raw- and vMonoCT image values are not necessarily linked to beam hardening alone. The linear attenuation coefficient measured through a uniform material will be different, albeit comparable, between a polyenergetic 60 kVp imaging beam and a virtual monoenergetic 35 keV imaging beam. Nevertheless, the goal of the percentage error maps is to emphasize the peripheral image gradients in the animal. In Figs. 3 and 4, an image intensity overestimation can be observed in the raw µCBCT image reconstruction, where the imaging beam traverses through a smaller part of the animal body. This phenomenon is emphasized and resolved by vMonoCT in the cranial region (black arrows in Fig. 4, Mouse #3 and #5), where the head of the animal was uplifted in the cone for the administration of anesthesia. Here, the vMonoCT prediction resulted in a µCBCT image intensity correction up to 15%.

In its current form, vMonoCT has two basic constraints compared to other published beam hardening resolving solutions^10,14,15,19^. First, rodents can be imaged with several preferred X-ray energy spectra ranging from 50 to 90 kVp. Therefore, it is stressed that vMonoCT was trained on projection data simulated with a 60 kVp polyenergetic X-ray beam, and 1.0 mm of aluminum spectral filtration. This configuration complies with the recommended X-ray imaging protocol on the SARRP device. Because tissue attenuation properties are energy dependent, it is evident that vMonoCT in its current form only applies to 60 kVp projection data of the SARRP device. However, the vMonoCT network architecture can be retrained separately with simulated projection data of another preferred energy spectrum (e.g., 80 kVp). Second, mice were picked as imaging subject, and although vMonoCT is expected to perform well on most mouse-sized subject due to the large degree of variation that was included in the training dataset, the network still needs to be scientifically validated for the imaging of other mouse strains.

In addition, a very tight time schedule is adopted in preclinical experiments because the mouse is physically constrained under anesthesia. All the workflow steps related to treatment planning of preclinical irradiations are recommended to take less than 20–60 minutes^34^, for reasons of workflow efficiency and animal well-being. Any additional step in the workflow should be well considered for cost-effectiveness and accuracy. Some empirical beam hardening correction algorithms demand a series of forward- and backward projections of the imaging data prior to a computationally intensive optimization process^14,15^, which is expected to be too time-consuming in preclinical practice. Here, the average application time of vMonoCT was recorded to be 18.8 s to predict virtual monoenergetic projections from 360 polyenergetic X-ray projections. In addition to the > 60 s acquisition and reconstruction times on the SARRP cabinet, a short prediction time of 53 ms per projection on a 16 GB T4 NVIDIA® GPU makes it worth to be implemented in the existing preclinical workflow. Especially because recent advances in the preclinical workflow already showed their enormous potential to reduce organ-at-risk segmentation times substantially^35,36^ or to enable automated beam-on time treatment planning for small animal radiotherapy^34^.

## Conclusion

In this work, a promising deep neural network architecture was developed to predict virtual monoenergetic µCBCT projections (vMonoCT). The vMonoCT network was trained on simulated monoenergetic and polyenergetic projection pairs of digital anthropomorphic mouse phantoms and mathematical volumes. Furthermore, the vMonoCT network performance was evaluated on one frozen mouse cadaver scan and on real retrospective 60 kVp projection data of five mice. The vMonoCT-based reconstructions showed a better µCBCT image uniformity than the conventional reconstructions, and the peripheral beam hardening artifacts and cupping artifacts were largely removed. Overall, these findings unveil the potential of deep learning algorithms to improve preclinical micro-CT image quality. It is anticipated from the study results that AI applications such as vMonoCT could play an important role in the technical efforts to improve the reproducibility and the quantitative consistency of preclinical mouse experiments that include high-resolution micro-CT imaging.

## Software availability statement

The software used in this study is made available online. https://github.com/brentvdh/vMonoCT.
